# Indole-3-Acetic Acid Biosynthesis by Endophytic Fungi and Its Association with Plant–Fungus Interactions

**DOI:** 10.3390/jof12050334

**Published:** 2026-05-04

**Authors:** Adiyadolgor Turbat, Aruna Vigneshwari, Thu Huynh, Nomuun Oyunbat, Apoorv Tiwari, Pramod W. Ramteke, Mónika Varga, Tamás Papp, Csaba Vágvölgyi, András Szekeres

**Affiliations:** 1 Laboratory of Microbiology, Institute of Biology, Mongolian Academy of Sciences, Peace Avenue-54b, Bayanzurkh District, Ulaanbaatar 13330, Mongolia; adiyadolgor_t@mas.ac.mn; 2 Department of Biotechnology and Microbiology, Faculty of Science and Informatics, University of Szeged, Közép Fasor 52, H-6726 Szeged, Hungary; arunabio2011@gmail.com (A.V.); huynh_thu@hcmut.edu.vn (T.H.); oyunbatnomuun@gmail.com (N.O.); varga.j.monika@gmail.com (M.V.); pappt@bio.u-szeged.hu (T.P.); csaba@bio.u-szeged.hu (C.V.); 3 Department of Biotechnology, Faculty of Chemical Engineering, Ho Chi Minh University of Technology (HCMUT), 268 Ly Thuong Kiet Street, District 10, Ho Chi Minh City 72607, Vietnam; 4 Vietnam National University Ho Chi Minh City, Linh Trung Ward, Thu Duc District, Ho Chi Minh City 71351, Vietnam; 5 Department of Computational Biology and Bioinformatics, Jacob Institute of Biotechnology and Bio-Engineering, Sam Higginbottom University of Agriculture, Technology and Sciences, Prayagraj 211007, Uttar Pradesh, India; apoorvbio@gmail.com; 6 Department of Biotechnology, Dr. Ambedkar College, Deekshbhoomi, Nagpur 440033, Maharashtra, India; pwramteke@gmail.com; 7 HUN-REN-SZTE Pathomechanisms of Fungal Infections Research Group, University of Szeged, Közép Fasor 52, H-6726 Szeged, Hungary

**Keywords:** phytohormone, plant growth promotion, IAA biosynthetic intermediates, stress tolerance, chemical messenger

## Abstract

Indole-3-acetic acid (IAA) is a central auxin regulating plant growth and developmental plasticity, including cell division, elongation, vascular differentiation, and root architecture. Beyond plants, many fungi—including endophytic fungi that reside within plant tissues without visible disease symptoms—can synthesize IAA and thereby modulate plant performance and plant–fungus interactions. However, fungal IAA biosynthesis remains less resolved than bacterial or plant pathways, and mechanistic evidence is uneven across taxa. This review summarizes recent (2020–2025) advances in IAA-producing endophytic fungi, the main proposed biosynthetic routes and intermediates, and methodological considerations for attributing IAA production to the fungal partner. The reported associations of fungal IAA with beneficial interactions are then discussed, including growth promotion, stress tolerance, and interaction-mediated changes in the root system’s architecture, with emphasis on the concentration- and context-dependent nature of plant responses. Overall, fungal IAA is associated with diverse plant–fungus interactions, as discussed in this review.

## 1. Introduction

Indole-3-acetic acid (IAA) is a major auxin that regulates key aspects of plant growth and development, including cell division and elongation, vascular differentiation (xylem and phloem), lateral root formation, and tropic responses to light and gravity [1,2,3]. Through these processes, IAA shapes root and shoot architecture and contributes to plants’ physiological and immune-related responses [4].

IAA was first identified as a plant-derived growth regulator; subsequent studies demonstrated that diverse microorganisms, including bacteria and fungi, can also synthesize IAA [5,6]. This discovery stimulated extensive research on microbial IAA and its roles in plant–microbe interactions [7]. Endophytic fungi (EFs) inhabit internal plant tissues without causing visible disease symptoms and can influence the host’s performance through multiple mechanisms, including the production of bioactive secondary metabolites and phytohormone-like compounds [8,9,10,11,12,13,14]. In addition to EFs, other plant-associated fungi—including members of rhizosphere communities and plant pathogens—are also capable of synthesizing IAA. Furthermore, certain free-living microorganisms (i.e., those that do not establish plant associations during their life cycle) can also produce this phytohormone [3].

In this review, the focus is placed on IAA produced by endophytic fungi. To this end, the literature search primarily covered peer-reviewed studies published between January 2020 and December 2025. Searches were conducted in Google Scholar, PubMed, and Scopus using combinations of keywords, including “endophytic fungi”, “fungal endophyte”, “indole-3-acetic acid”, “IAA biosynthesis”, “auxin”, and “plant–fungus interaction”. Earlier studies were selectively included where they provide foundational insights into biosynthetic pathways or widely applied methodologies.

The ecological niche of endophytes—characterized by sustained metabolic exchange with the host—can exert strong evolutionary pressure and may promote the diversification of fungal secondary metabolites with novel biological activities [15]. Plants may benefit from EF-derived phytohormones and related metabolites that contribute to growth and development [16]. In return, the host provides nutrients such as carbohydrates and amino acids, supporting a mutualistic lifestyle often described as plant growth-promoting fungi [17]. In this context, fungal IAA has been implicated in host colonization and in modulation of plant growth responses [18]. Importantly, elevated IAA levels are not universally beneficial for plants, as several plant pathogenic fungi are also capable of synthesizing IAA, where it may function as a virulence or pathogenicity-related factor. Thus, the effects of IAA are highly context-dependent and may vary, depending on its concentration, source, and the nature of the plant–microbe interaction [19]. Consequently, IAA-producing endophytes are of interest for developing environmentally friendly microbial inoculants and biofertilizer formulations in sustainable agriculture [17].

This review summarizes (i) endophytic fungal taxa reported to produce IAA, (ii) proposed biosynthetic routes and intermediates in fungi, and (iii) evidence linking fungal IAA production with plant–fungus interactions, while highlighting methodological limitations and key knowledge gaps for future research.

## 2. The Biodiversity and Distribution of IAA-Producing Endophytic Fungi

EFs occur in diverse hosts, ranging from algae and bryophytes to ferns and seed plants, and have been reported across habitats from tropical and temperate regions to arctic, alpine, aquatic, and xerophytic ecosystems [20]. Accordingly, IAA-producing endophytic fungi have been reported from a wide range of host plants and geographic regions (Table 1) [21,22,23,24]. IAA production has been detected both in the presence and absence of L-tryptophan (Trp); however, in many studies, Trp supplementation increased IAA yields in culture, although strain- and condition-specific exceptions have been reported [18,21,25,26,27].

Several EF isolates have been reported to produce IAA in axenic culture, frequently with increased yields upon Trp supplementation (Table 2) [18,21,25,26,27,30,33,34]. For example, *A. sorghi* and *P. commune* isolated from common bean produced IAA in Trp-supplemented broth [25], and *A. niger* associated with Purpus’ carpetgrass showed markedly higher IAA yields under higher Trp availability [21]. Similar Trp-associated increases have been observed for *C. fructicola* from arabica coffee and *C. geniculata* from congress grass [26,27]. In addition, stress conditions may modulate IAA output; for instance, *Penicillium funiculosum* increased IAA production under combined heavy metal stress [35] (Table 2).

Medicinal plants are great reservoirs of EFs that are believed to be involved in the production of pharmaceutical products including IAA [22]. *Cyanodermella asteris* associated with Tatarian aster (*Aster tataricus*) (a traditional Chinese medicine with antibacterial, antiviral, antiulcer, and diuretic activities) was reported to synthesize IAA under precursor-free conditions and in assays supplemented with proposed intermediates (indole-3-acetonitrile (IAN), indole-3-acetamide (IAM), and indole-3-acetaldehyde (IAAld)). In these experiments, *C. asteris* secreted most of the IAA into the medium (64% to 92%) and retained only small amounts in its hyphae (8% to 36%) [38].

Jerusalem artichoke (*Helianthus tuberosus*)*,* Siam weed, ginger, and wild asparagus (*Stemona tuberosa*) are well-known traditional herbs with antifungal, antioxidant, and anticancer activities. *M. phaseolina* and *Diaporthe phaseolorum* isolated from Jerusalem artichoke, *M. phaseolina* isolated from Siam weed, *T. koningii* isolated from ginger, and *Daldinia eschscholtzii* isolated from wild asparagus were reported as IAA producers. Remarkably, *M. phaseolina* isolated from Jerusalem artichoke exhibited higher IAA-producing ability than others using indole-3-lactic acid (ILA) and indole-3-acetamide (IAM) routes [22]. The medicinal herb Sinai ephedra is highly adapted to arid and semi-arid habitats. An endophytic *P. commune* isolate from Sinai ephedra could produce a maximum IAA productivity in a culture with Czapek–Dox broth containing 5 µg/mL Trp at 28 °C for 14 days (Table 2) [31]. Endophytic *P. simplicissimum, Talaromyces flavus*, and *T. konilangbra*, isolated from the roots of stevia (*Stevia rebaudiana*), Dahurian angelica (*Angelica dahurica*), and Ming Aralia (*Polyscias fruticose*) in Vietnam, respectively, were cultured under IAA-inducing conditions (20 g/L peptone; 1.15 g/L K_2_HPO_4_; 1.5 g/L MgSO_4_; 1.5% (*v*/*v*) glycerol; 0.1 g/L Trp) for 48 h at 30 °C [24]. Fifteen strains of *Alternaria, Didymella, Fusarium*, and *Xylogone* isolated from shrubby sophora (*Sophora flavescens*) (a medicinally important plant in Mongolia and China) produced IAA in PDB broth at 25 °C for 7 days without or with 0.1 g/L Trp. However, IAA production generally improved due to the additional Trp [39].

Orchids are obligately mycorrhizal plants and represent one of the largest angiosperm families [18,29,36]. Eight isolates (including *Penicillium*, *Fusarium*, *Coniochaeta*, *Alternaria*, and *Cladosporium*) obtained from long-horned dendrobium were reported to produce IAA in culture [18]. Thirteen isolates of *C. gloeosporioides*, *Eupenicillium* sp., *Fusarium* sp., *Tulasnella* sp., and *Trichocladium pyriforme* associated with Thai native terrestrial orchids (similar spathoglottis (*Spathoglottis affinis*), egg-in-a-nest orchid (*Paphiopedilum bellatulum*), and nun’s-hood orchid (*Phaius tankervilleae*)) synthesized IAA in culture with PDB supplemented with various concentrations of Trp (0, 1, 2, 4, 6, 8, and 10 mg/mL) and incubation in dark on a shaker at 25 °C for 14 days. The maximum yield of IAA was revealed when samples were cultured with 4 mg/mL of Trp and then decreased when the concentration of Trp reached 6 mg/mL (Table 2) [36]. Shihu, an ornamental and medicinal orchid in the central hills of Nepal, is a typical epiphytic and deciduous species requiring less water and nutrients to grow. Nine isolates, including *Cladosporium*, *Colletotrichum*, *Cylindrocarpon*, *Fusarium*, *Hypoxylon*, *Leptosphaerulina*, and *Trichoderma*, from this orchid could synthesize higher IAA concentrations in broth supplemented with 1 mg/L of Trp compared with cultures without Trp [29]. Twelve EF isolates obtained from the wild orchid comb vanda (*Vanda cristata*) promoted the growth of aloe-leafed cymbidium (*Cymbidium aloifolium*) protocorms, and their IAA production increased with Trp concentration (1 < 2 < 5 mg) [23].

Drought is a severe abiotic stress that threatens plant growth and crop productivity and is expected to intensify under climate change. *Aspergillus awamori* was isolated from the leaves of drought-stressed ashwagandha (*Withania somnifera*), a well-known medicinal plant in India. The IAA-producing ability of the fungal strain was determined in Czapek–Dox broth containing various concentrations of Trp (0, 0.1, 0.5, 1 mg/mL) at 28 °C for 7 days. The production of IAA was 27% and 40% greater in cultures containing 0.5 and 1 mg/mL of Trp [40]. *A. alternata*, isolated from Dahurian wildrye (*Elymus dahuricus*), produced IAA under both Trp-supplemented and Trp-free conditions; however, the IAA yield increased markedly with Trp supplementation [41]. Desert habitats experience extreme environmental conditions such as drought, extreme light exposure, high temperature, extreme pH, and salinity. The *Sordariomycetidae* sp. CJAN1179 strain isolated from the perennial grass Iwarancusa grass in Cholistan Desert (Table 1) is a mutualistic fungus that promotes plant growth by producing IAA and mediates lateral root growth. This strain synthesized IAA along with tryptophol (TOL) as a by-product associated with the indole-3-pyruvic acid (IPyA/IPA) route of the Trp-dependent pathway [32]. Felty germander is a medicinal plant living in the extremely arid climate of Saint Katherine Protectorate, Sinai Peninsula, in Egypt (Table 1). *P. chrysogenum* and *A. alternata* isolated from Sinaian milkweed (Table 1) could produce IAA and enhance the root growth of maize plants. Regarding Trp as a precursor of the IAA production, *P. chrysogenum* and *A. alternata* increased the yield of IAA with the increase in Trp concentration in growth broth. The significant variation in their IAA production was in the culture with Czapek–Dox broth at 28 °C for 10 days at concentrations of 1, 2, and 5 mg/mL of Trp; the IAA-producing result at the concentration of 5 mg/mL Trp was doubled compared with that without Trp [28].

Salinity is a major abiotic stress that disrupts plant growth and metabolism by imposing ionic and osmotic stress and altering phytohormone balance [37]. Climate change is expected to exacerbate salinity stress in many agricultural regions [42]. *Aspergillus ochraceus* isolated from leaves of grey mangrove (*Avicennia marina*) in a semi-arid environment exhibited high salt tolerance (up to 200 g/L NaCl) and mitigated seawater-related stress effects in barley. IAA production in culture was higher at 30% seawater than at 15% seawater, and Trp supplementation further increased yields [43]. *P. formosus* from the roots of cucumber plant produced IAA and enhanced the growth of Waito-C and Dongjin-byeo rice seedlings as well as cucumbers under saline stress [37]. *Phoma glomerata* isolated from host cucumber plants could produce IAA without or with 0.1 g/L Trp in Czapek medium incubated at 30 °C for 7 days and promoted cucumbers’ growth under saline and drought stress [44].

## 3. Biosynthesis of IAA in Different Fungi

As mentioned above, both Trp-dependent and Trp-independent IAA biosynthetic pathways coexist in plants [26,45] as well as in microorganisms [46]. However, previous studies were more focused on Trp-dependent IAA biosynthesis and only a few articles can be found on Trp-independent pathways (Table 3). However, the evidence for truly Trp-independent IAA biosynthesis in fungi is limited, and pathway assignments are often indirect. Despite the fact the physiological character of IAA in plants is well described and more IAA biosynthesis pathways have been proposed, only a limited number of biosynthesis genes have been identified or functionally validated in fungi [1,47]. In most described microbial routes, Trp serves as the primary precursor for IAA biosynthesis [46]. The pathways are further referred to according to the major metabolite produced downstream of Trp. It has been documented that within the microorganisms, both bacteria and fungi are capable of synthesizing IAA [3,17,48] and may operate in similar ways to those found in plants, but there are also different pathways [47,48].

On the other hand, IAA biosynthetic pathways in bacteria and plants have been widely studied, whereas few studies on the IAA biosynthetic pathway in fungi have been performed until now [38,51,53,59]. The mechanism, specifically how fungal endophytes synthesize IAA, is crucial to finding out the communication between EFs and their host plants [17,61,62]. IAA production in different EFs has been recorded and proved to be dependent on the main precursor, Trp [3,48,59,63].

**Figure 1:**
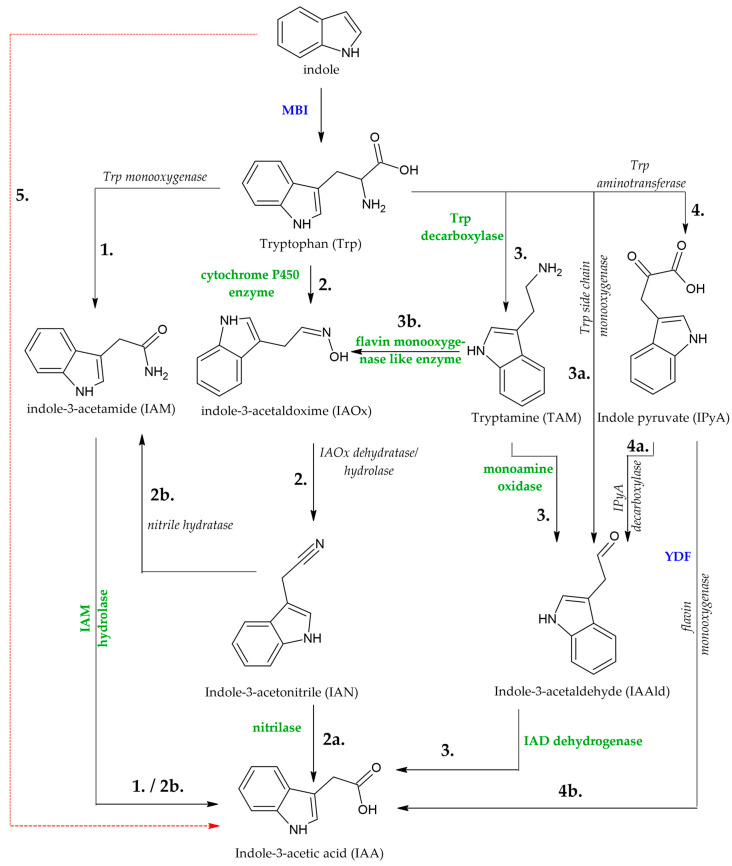
Summary of IAA biosynthesis pathways in bacteria, fungi, and plants with their known enzymes [38]. IAA can be transformed from Trp (Trp-dependent pathways, 1–4). Putative non-tryptophan routes have been proposed in microorganisms, but mechanistic evidence—particularly in fungi—remains limited; therefore, these steps are shown to be hypothetical where applicable (Trp-independent pathways are with the dashed red line, 5). Bacteria and fungi mostly prefer the IAM (1), the IAOx–IAN (2-2a or 2-2b), the TAM (3), or the IPyA (4-4a-3 or 4-4b) pathways. The IAA pathway with the direct conversion of Trp to IAAld, without IPyA as an intermediate, 3a-3 [5]. The reaction site of the IAA biosynthesis inhibitors 2-mercaptobenzimidazole (MBI) (Trp synthase) and yucasin DF (YDF) (YUC enzymes) is shown in blue. Enzymes for the candidate genes are highlighted in green.

To date, five IAA biosynthetic routes have been described in microorganisms, which are the IPA, IAM, IAN, tryptamine (TAM), and Trp sidechain oxidase (TSO) pathways [46,64,65]. In bacteria, the IAM, IAN, and IPyA/IPA routes are among the most commonly discussed IAA biosynthesis pathways and have been described across many plant growth-promoting bacterial species [3,46,51,56,59,66,67,68]. Regarding fungi, the IPA pathway is most frequently mentioned for IAA biosynthesis [51,53,69,70], but the TAM and IAM routes have been also noted [51,53] (Table 3). In many studies, pathways have been inferred from the detection of IAA and putative intermediates in culture extracts (Figure 1). However, metabolite detection alone does not prove pathway operation, and genetic/enzymatic validation is required for definitive assignment. Accordingly, the presence of IAM, IPyA/IPA, TAM, IAAld, IAN and related compounds has been used as *indicative* evidence for IAM-, IPyA/IPA-, TAM-, TSO-, or IAN-associated routes, although overlapping metabolite profiles can complicate interpretation [46,49,71]. As mentioned, certain intermediates of IAA biosynthesis can be converted into storage compounds (Figure 1), e.g., the reduction of IPA and IAAld to ILA and indole-3-ethanol (IOL) or TOL [49]. The physiological function of these compounds remains unclear.

The early step of the IPA pathway starts with Trp’s conversion to IPA through aminotransferase activity, and then IPyA/IPA is decarboxylated to IAAld via indole-3-pyruvate decarboxylase (IPDC) (Figure 1). The final step is the oxidation of IAAld to IAA [5,71]. In spite of that, some intermediates, namely IPA and IAAld, could be reversibly transformed into storage compounds. In view of this fact, IOL and ILA were described as stable compounds and efficiently transformed from IPA and IAAld [46,72]. This IPA pathway was found in *U. maydis* and the enzymes involved were identified, namely the Trp aminotransferases (TAMs) Tam1 and Tam2, which participated in the conversion of Trp into IPA [59,73], and the IAAld dehydrogenases, Iad1 and Iad2, converting IAAld to IAA [53].

Downstream of IPA in *U. maydis*, the IAAld dehydrogenases (UmIAD1 and UmIAD2) also convert IAAld into IAA [59]. Similarly, the phytopathogenic fungus *L. maculans* used IPDC (LmIPDC2) to catalyze conversion from IPA to IAAld for IAA biosynthesis [56].

Until now, *U*. *maydis* and *Tricholoma vaccinum* were the only fungal species reported from which IAA metabolic genes from the IPA pathway have been isolated and functionally identified [60]. Consequently, Trp-dependent IAA biosynthesis proposes that the IAM pathway is devoted to IAA biosynthesis at an earlier stage; however, the IPA pathway may be activated only later [51]. Hence, IAM accumulated as an intermediate from Trp supplementation, and this illustrates marked differences from *U. maydis*, in which adequate conversion of Trp to IPA and of IAAld to IAA arises by distinguishing no or low amounts of IPA in culture filtrates [74].

The root endophyte *Serendipita indica* (formerly *P. indica*) forms beneficial associations with a range of host plants, including crops such as barley, wheat, and maize, and has been reported to produce IAA [75]. *S. indica* has also been noted to produce IAA and ILA via IPA as an intermediate compound. Disruption of a Trp aminotransferase gene in *S. indica* resulted in a marked reduction in IAA and ILA production [57]. In some *Fusarium* species, evidence has also supported IAM-associated IAA biosynthesis, indicating that more than one Trp-dependent route may operate, depending on the species and conditions [3,53]. According to the study, TOL was detected as a specific by-product of the IPA pathway. The IAM route is often reported as the second most common Trp-dependent pathway in endophytic fungi (Figure 1). In the case of the IAM pathway, this pathway has two steps. Trp is first transformed to IAM via the IaaM enzyme, and the next step is IAM’s conversion to IAA through IaaH activity [5,46]. In *Fusarium*, the IAM pathway was observed, where IaaM and IaaH also act in IAA biosynthesis [3]. Therefore, some indole intermediates were discovered, such as IAA, IAM, TOL, ILA, and indole-3-carboxylic acid (ICA). In addition, the presence of these intermediates in the culture implied that two IAA biosynthetic pathways that use Trp as a precursor may exist in *C. gloeosporioides* f. sp. *aeschynomene* [51].

The fungal endophyte *C. fructicola* synthesizes IAA via the IAM pathway, which was detected through the IaaM activities of fungal IAA production; meanwhile, some EFs like *C*. *gloeosporioides*, *F. proliferatum*, *F*. *verticillioides*, *F*. *fujikuroi, F*. *oxysporum, F. armeniacum*, *Did. glomerata*, *A. ternuissima*, and *f.* sp. (SZMC 26660) use the IAM pathway for their IAA synthesis [3,49,50,51]. Also, in *C. gloeosporioides*, IAM played a role in dynamic IAA formation, while IAAld was chiefly converted to TOL with minor IAA formation. To date, this was the first time that the IAM pathway was discovered in fungi. Contrasts were recognized in the expressions of the two pathways during the growth of the fungus, and distinctions in the activity of the IAM and IPA pathways were newly described in *E. herbicola* [50] and also the IAM pathway reported in *C. acutatum* [51,52].

The IAM pathway proceeds through IAM and, in the case of the IPA pathway, is affected through TOL and ILA [5] (Figure 1). During an experiment, IAA accumulated to high levels when IAM was added to the medium; at the same time, the IAM levels weakened with time, showing that IAM is absolutely imperative for IAA biosynthesis in *C. gloeosporioides* [51]. The detection of IaaM and IAM hydrolase’s enzymatic activities as well the detection of IAM in infected plants proved that *C. gloeosporioides* f. sp. *aeschynomene* not only communicates with the IAA biosynthesis genes but also conducts IAM and IAA in planta, as Virginia jointvetch (*Aeschynomene virginica*) plants do not maintain these enzymatic activities and are inadequate for synthesizing IAM [50]. In addition, *U. esculenta* and *U. scitaminea* were able to produce IAA from Trp; however, IAM was not distributed as a precursor compound. The IAM pathway confirmed, for the first time, that *Didymella* and *Alternaria* species conducted IAA synthesis through the IAM pathway [49].

Regarding the IAN pathway, the intermediates of the IAN pathway are indole-3-acetaldoxime (IAOx), IAN, and IAM (Figure 1). This pathway starts from Trp then it is converted to IAOx via glucobrassicin and then transformed into IAN. From here, it converts to IAM via nitrile hydratase activity and finally, it is converted to IAA by IAM hydrolase activity. Measurement of IAN or both IAN and IAM detected together in the samples mark the function of the IAN pathway [5,71]. Numerous fungal endophytes function through the IPA and IAM pathways for producing IAA, but the IAN pathway has only been discovered in two fungal endophytes, *F. graminearum* and *S. sclerotiorum* [54,55].

During the TAM pathway, the first step is decarboxylation of Trp to TAM, which is then converted to IAAld by amine oxidase (Figure 1). As the last step, IAAld is transformed into IAA. It was reported that a few fungal endophytes were able to conduct IAA biosynthesis through the TAM pathway such as the insect pathogenic fungus *M. robertsii*, Trp decarboxylase has been noted. Furthermore, *F. graminearum* was also able to convert TAM into IAA [55]. Moreover, within the strains isolated from shrubby sophora, *Fusarium* (SZMC 26983, 26990, 27017, and 27037) and two *Didymella* strains (SZMC 26648 and 26991) were able to produce IAA from TAM [49]. Another study states that the fungal endophyte *C. asteris* synthesized IAA by different pathways, and one of these pathways is the TAM pathway, as all necessary genes were found in that microorganism [38].

The TSO pathway includes steps where Trp is directly converted to IAAld prior to the oxidation of IAAld to IAA, while intermediates could be directly converted to IAA by nitrilase or transformed into IAM prior to conversion of IAM to IAA by nitrile hydratase in the IAN pathway [5,71]. Hence, the fungus *Alternaria* sp. (SZMC 26985) utilized the TSO pathway for IAA biosynthesis. In addition, in the fungal endophytes *Alternaria* sp. (SZMC 26986) and *Phoma* sp. (SZMC 27036), as a by-product of IAAld, the presence of only TOL was found, which could have originated from the IPA and TAM pathways, but also from TSO pathway due to the loss of other indicator metabolites. Moreover, besides the IPA and TAM pathways, the existence of the TSO pathway could not also be excluded in these isolates due to the overlapped intermediate profiles of these pathways [49]. Interestingly, it was indicated that some fungal endophytes implement two or more biosynthetic pathways for producing IAA [49,52]. It is important to point out that the IAM, IPA, and TAM pathways co-exist in *Fusarium* species (SZMC 26990, 27017, and 27037). EFs were capable of synthesizing IAA through three pathways, including the pathway pairs IPA–TAM in *Didymella* species (SZMC 26648 and 26991) and IPA–IAM in *Fusarium* species (SZMC 26654 and 26979), and IAM–TAM in *Fusarium* species (SZMC 26983) [49].

Besides Trp-dependent biosynthetic pathways, there is confirmation that IAA can be produced in other biosynthetic ways. In *Saccharomyces cerevisiae*, evidence has been interpreted to suggest Trp-independent IAA formation based on mutant analyses and radiolabeling experiments [76]. Another study reported that *T. virens* interacts with Arabidopsis (*Arabidopsis thaliana*), and fungal IAA biosynthesis may contribute to this interaction [62]. Once the EF produced IAAld and IOL in addition to IAA, it can be simulated that at least the IAAld pathway is active in this fungal endophyte. On the other hand, the arbuscular mycorrhizal fungi *Rhizophagus irregularis* was considered for the presence of IAA production, but no biosynthetic pathway has been indicated up to now [74]. Numerous fungal endophytes such as *Alternaria* sp. (SZMC 26975, SZMC 27004, SZMC 27018, SZMC 26651, and SZMC 26653), *C*. *acutatum*, *Did. glomerata* (SZMC 27019 and SZMC 26655), *F. chlamydosporum* (SZMC 27000), *F. proliferatum*, *F*. *fujikuroi*, *F*. *oxysporum*, *Muscodor cinnamomi*, *Magnaporthe oryzae, P. chrysanthemicola* (SZMC 27034), *P. chrysogenum* (SZMC 26987), *S. indica*, *U. esculenta*, and *U*. *maydis* primarily act through the IAM and IPA pathways for IAA biosynthesis [3,49,51,57,59,66,77].

Although genomic resources for many fungi are available, detailed functional annotation and validation of IAA biosynthesis-related genes remain limited. The enzymes involved are aldehyde dehydrogenase, peroxisomal copper amine oxidase, 3-hydroxyphenylacetate-6-hydroxylase, nitrilase, cysteine sulfinic acid decarboxylase, hypothetical protein, aromatic amino acid aminotransferase, pyruvate decarboxylase, amino transferase, hypothetical protein, kynurenine/alpha-aminoadipate aminotransferase, hypothetical protein, succinyl-CoA ligase subunit beta, and glutamyl-tRNA amidotransferase, and there are several hypothetical proteins (Figure 1). The role of these genes was described in the IPA pathway contributing to IAA production by generating a single deletion mutant of each gene, and it was found that the IAA content was significantly reduced [78].

## 4. Role of Fungal IAA in Biological Processes

### 4.1. Fungal IAA in Plant Growth-Related and Signaling Contexts

Increasing evidence links fungal IAA production with plant growth-related responses. Comparatively, higher growth was observed in the plants colonized by IAA-producing EFs compared with those without IAA-producing fungi [34,35,55,77,79]. However, numerous studies associate fungal IAA production with plant growth promotion, but it is important to note that excessive IAA levels may have detrimental effects, including growth inhibition or altered plant development. Moreover, in plant–pathogen interactions, fungal-derived IAA has been implicated in the modulation of host physiology in ways that may facilitate infection [19].

Culture extracts from seven IAA-producing EFs (*Aspergillus oryzae, Aspergillus terreus, Curvularia* sp., *F. oxysporum*, *Gliocladium solani*, *P. citrinum,* and *T. viride*) improved root growth in maize seedlings [79]. The production of IAA by the EF *F. oxysporum* has been reported to promote maize growth in hydroponic environments [62]. Another study showed that the EFs *P. chrysogenum* (Pc_25) and *A. alternata* enhanced the root growth of the plant Sinaian milkweed [28]. IAA-producing EFs such as *Alternaria, Didymella*, *Fusariumi*and *Xylogone* isolated from shrubby sophora were examined in plant bioassays. In these tests, primary root length increased in several cases, whereas biomass was significantly lower than in the IAA-treated control [49]. IAA production by *A. niger* was associated with plant growth-promoting effects in common bean, Carioca cultivar [21]. A culture broth of *Chaetomium globosum* increased cucumber growth (radicle length, plant height, root length, and biomass), consistent with efficient colonization and phytohormone production, including IAA [34]. In addition, EF isolates from Shihu were reported to produce IAA and to promote germination and early growth of orchid plantlets and protocorms (aloe-leafed cymbidium and long-horned dendrobium, respectively) under in vitro conditions [18]. A root EF (*Sordariomycetidae* sp. CJAN1179) isolated from Iwarancusa grass increased lateral root formation in Arabidopsis, potentially via fungal signals that modulate plant auxin-related pathways during interaction [32]. The increase in IAA produced by the fungal endophyte *Diaporthe terebinthifolii* was linked to a significant increase in glycyrrhizin content in licorice plants, highlighting the role of IAA-producing fungi in stimulating plant growth and secondary metabolite production. This suggests that IAA may be involved, directly or indirectly, in regulating the pathways that lead to glycyrrhizin synthesis in licorice [80].

Plant growth can be influenced positively or negatively by the interplay between endophytic fungi and fungi in the rhizosphere. This synergism can be used to promote the growth of hosts plants as well as fungal species that interact with one another [81]. According to the research by Suebrasri et al., the EFs *D. phaseolorum* and *M. phaseolina*, which were isolated from Siam weed and ginger, are able to produce the IAA hormone and boost sunchoke plants’ growth [22]. These results suggest that co-cultivation may influence IAA production, which could be relevant for future agricultural applications [18].

Recent research has highlighted the importance of fungal IAA as a signaling molecule in plant–microbe interactions, including fungal pathogenesis and mycorrhizal associations. A study identified the EF *A. awamori* from leaves of ashwagandha and screened it for secondary metabolites. They found that the strain was producing IAA, and this isolate enhanced the growth of the host plant and efficiently colonized in maize plants. When the biosynthesis of IAA in leaves and roots was inhibited, colonization efficacy was reduced by 52% and 66%, respectively. Interestingly, the restoration of IAA biosynthesis again enhanced the colonization of *A. awamori* in maize roots and established beneficial endophytic associations, supporting the view that IAA may contribute to signaling during plant–fungus interactions [40].

Mycorrhizal associations are well-studied examples of mutually beneficial plant–fungus symbioses and share functional similarities with endophytic plant–fungus inter-actions. In ectomycorrhizae (e.g., *T. vaccinum*), IAA has been reported to act as a diffusible signal, influencing root colonization and promoting Hartig net formation. The role of IAA as a signaling molecule in plant–fungus interactions is further supported by the identification of interaction-induced, mycorrhiza-specific genes involved in IAA production and transport [82].

The effect of the mycorrhizal fungi *Funneliformis mosseae* on IAA level and indole acetic acid oxidase activity was also evaluated, as well as the expression of IAA-related genes under 0.1 mM and 1.0 mM of phosphorous to study the effect on root hairs. IAA concentrations were found to be higher in mycorrhizal seedlings, and their root hair density was higher compared with non-mycorrhizal seedlings. It has been found that indole-3-pyruvate monooxygenase and auxin transporter-like protein 3 expression levels in roots were significantly upregulated by mycorrhizal treatment, which indicated that mycorrhizal colonization induced the IAA accumulation in roots [83].

### 4.2. Fungal IAA in Plant Defence Mechanisms

Fungal-produced IAA has been associated with systemic defense-related responses against phytopathogens. It is important to note that several fungal species discussed in this review may also exhibit pathogenic or opportunistic lifestyles. For example, *Fusarium*, *Alternaria*, *Colletotrichum*, and *Magnaporthe* species are well-known phytopathogens causing significant crop losses worldwide [19], while *Aspergillus* and *Fusarium* species are recognized as opportunistic human pathogens and potential mycotoxin producers [84]. In such cases, IAA production cannot be interpreted solely as a beneficial trait, as it may also contribute to pathogenicity or host manipulation. Therefore, the role of fungal-derived IAA should be considered in a context-dependent manner, taking into account the ecological function and lifestyle of the producing organism.

The use of IAA-producing *Trichoderma* in a pre-treatment of tomato plants showed the production of defense related enzymes against the aerobic non-spore-forming, Gram-negative, plant pathogenic bacterium *Ralstonia solanacearum* [85]. Studies have indicated that IAA could reduce pathogen infection by altering the gene expression and homeostasis of antioxidants [86]. An investigation on IAA-producing EFs *Curvularia lunata*, *Pholiota multicingulata*, *T. atroviride*, *T. harzianum*, and *S. commune* isolated from healthy rhizome of *Elettaria* sp. in Subayak forest, North Sumatra, reported that they showed antagonistic activity towards the fungal plant pathogens *Rigidoporus lignosus* and *Ganoderma boninense* which cause white root rot [87]. Another study proved that the three IAA-producing EFs were active against five plant phytopathogenic fungi (*F. oxysporum*, *Sclerotium rolfsii*, *Rhizoctonia* sp., *Alternaria brassicicola*, and *Phytophthora palmivora*) [88]. Another interesting study shed light on the potential virulence effect of fungal IAA on insects. It has been observed that the entomopathogenic fungus *M. robertsii* could produce IAA and enhance plant growth and stimulate virulence against insects, suggesting that entomopathogenicity may also be influenced by endogenous IAA biosynthesis. Several insect pathogens, including *Metarhizium* species and *Beauveria bassiana*, have also been reported to produce IAA, suggesting that interactions between plant- and fungal-derived IAA may play an important role in plant–microbe–insect interactions [55].

Notably, several phytopathogenic fungi are able to produce IAA, and in such cases, IAA has been considered a potential pathogenicity factor, contributing to host tissue colonization or suppression of plant defense responses [19].

### 4.3. Fungal IAA in Plant Stress Tolerance

Heavy metal-contaminated soils are a serious environmental issue that harms agriculture and the ecosystem. Globally, economic and efficient solutions are required to address this issue. In this context, extensive research is required to investigate the use of suitable microbial strains that can promote crop growth in heavy metal-contaminated soils. Fungal IAA has been shown to improve stress tolerance in a variety of plants under various environmental stress conditions [35,43,89]. A study on wheat plants grown in heavy metal-enriched soil revealed that the inoculation of IAA-producing EF *P. roqueforti* inhibited the transfer of heavy metals such as Ni, Cd, Cu, Zn, and Pb to plants and resulted in higher growth, whereas stunted growth was observed in the control group. These results support an association between IAA-producing EFs and enhanced heavy metal phytostabilization [89].

In other research, the *T. longibrachiatum* strain effectively promoted wheat growth and enhanced plants’ tolerance to NaCl stress through increased 1-aminocyclopropane-1-carboxylate (ACC) deaminase activity and IAA production and regulated the transcriptional levels of IAA and ethylene synthesis genes’ expression in wheat seedling roots under salt stress. The growth of the wheat seedlings was aided by these biochemical, physiological, and molecular responses, which also improved the plants’ resistance to salt stress [90]. IAA produced by *A. niger* improved the growth of maize seedlings under high NaCl and KCl stress [91]. Additional evidence suggested an association between fungal IAA production and enhanced plant development under salinity, cold, and heat stress [92]. A study on the mycorrhizal fungus *F. mosseae* proved that the mycorrhizal colonization enhanced endogenous hormone changes, especially IAA, resulting in increased root hair development and improved drought adaptation of trifoliate orange *(Poncirus trifoliata*) seedlings [83]. Another fungus, *P. funiculosum*, was able to enhance also the growth of soybean under metal stress [35]. *P. formosus* isolated from cucumber roots produced IAA and enhanced the growth of rice seedlings (Waito-C and Dongjin-byeo) and cucumbers under saline stress [37].

Although growing evidence suggests that IAA plays a role in establishing symbiotic relationships with plants and increasing stress resistance, more research is needed to fully understand the mechanism. Recent syntheses and mechanistic summaries provide additional context [16,93].

## 5. Conclusions

Plants engage in multifaceted interactions with the diverse microbes present in their ecosystems. Accumulating evidence suggests that indole-3-acetic acid (IAA) may act as an important signaling molecule in plant–microbe communication. Taken together, the role of fungal IAA in plant–fungus interactions is complex and context-dependent, as it may contribute to both beneficial and detrimental outcomes, depending on the ecological and physiological context. Genomic and transcriptomic studies have substantially advanced our understanding of IAA-associated signaling in bacteria; however, dedicated functional genomic and biochemical studies are still needed to clarify how IAA contributes to plant–fungus communication. Diverse fungal taxa can produce IAA via multiple biosynthetic routes, but the underlying molecular mechanisms and their links to fungal physiology, colonization strategies, and (in some cases) pathogenicity remain incompletely understood. Addressing these gaps will be essential for the robust and safe exploitation of IAA-producing fungi as sustainable agricultural inoculants Taken together, current evidence supports an association between fungal IAA production and plant–fungus interactions, while emphasizing the complexity and context-dependent nature of these processes.

## Figures and Tables

**Table 1:** Endophytic fungal species reported to produce IAA, their sources, and host plants.

IAA-Producing EF	Host Plant	Origin	Reference
*Alternaria alternata*	Sinaian milkweed (*Asclepias sinaica*)	Desert; 28°32′36″ N, 33°55′59″ E—Ain Shakaya, Saint Katherine, South Sinai, Egypt	[28]
*Alternaria sorghi*	Common bean (*Phaseolus vulgaris*)	30°38′34″ N, 30°56′54″ E—El-Menofia governorate, Egypt	[25]
*Alternaria* sp.	Long-horned dendrobium (*Dendrobium longicornu*)	Forest; 27°36′29″ N, 85°5′39″ E—Chitang, Makwanpur, the central hills of Nepal	[18]
*Aspergillus niger*	Purpus’ carpetgrass (*Axonopus purpusii*)	Nhumirim farm; 18°59′0″ S and 56°39′0″ W—Embrapa Pantanal, Nhecolândia sub-region of Pantanal, Corumbá City, MS, Brazil	[21]
*Cladosporium* sp.	Long-horned dendrobium (*D. longicornu*)	Forest; 27°36′29″ N, 85°5′39″ E—Chitang, Makwanpur, the central hills of Nepal	[18]
*Colletotrichum alatae*	Shihu (*Dendrobium moniliforme*)	forest; 27°36′29′′ N, 85°50′39′′ E—Daman, Makwanpur District, Nepal	[29]
*Colletotrichum fructicola*	Arabica coffee (*Coffea arabica*)	18°50′43″ N, 98°54′26″ E—Khun Changkean of Highland Research and Training Center, Faculty of Agricultural, Chiang Mai University, Thailand	[26]
*Coniochaeta* sp.	Long-horned dendrobium (*D. longicornu*)	Forest, 27°36′29″ N, 85°5′39″ E—Chitang, Makwanpur, the central hills of Nepal	[18]
*Curvularia geniculata*	Congress grass (*Parthenium hysterophorus*)	Bharathiar University campus; 11°2′24″ N, 76°52′48″ E—Coimbatore, Tamilnadu, India	[27]
*Fusarium* sp.	Long-horned dendrobium (*D. longicornu*)	Forests; 27°36′29″ N, 85°5′39″ E—Chitang, Makwanpur, the central hills of Nepal	[18]
*Macrophomina phaseolina*	Siam weed (*Chromolaena odorata*)	16°27′38′′ N, 102°49′4′′ E—Khon Kaen, Thailand	[22]
*Penicillium chrysogenum*	Felty germander (*Teucrium polium*)	Desert, 28°32′21″ to 28°32′21″ N, 33°55′51″ to 33°55′14″ E—Wadi al-Zwatin, Saint Katherine Protectorate, Sinai Peninsula, Egypt	[30]
*P. chrysogenum*	Sinaian milkweed (*A. sinaica*)	Desert, 28°32′36″ N, and 33°55′59″ E—Ain Shakaya, Saint Katherine, South Sinai, Egypt	[28]
*Penicillium commune*	Sinai ephedra *(Ephedra pachyclada*)	Wadi Selebat; 28°32′44″ to 28°32′36″ N, 33°56′1″ to 33°55′59″ E—the World Heritage site of Saint Katherine, South Sinai, Egypt	[31]
*P. commune*	Common bean (*P. vulgaris*)	El-Menofia governorate; 30°38′34″ N, 30°56′54″ E—Egypt	[25]
*Penicillium crustosum*	Felty germander (*T. polium*)	Desert; 28°32′21″ N, 33°55′51″ to 33°55′14″ E—Wadi al-Zwatin, Saint Katherine Protectorate, Sinai Peninsula, Egypt	[30]
*Penicillium* sp.	Long-horned dendrobium (*D. longicornu*)	Forest; 27°36′29″ N, 85°5′39″ E—Chitang, Makwanpur, the central hills of Nepal	[18]
Unidentified member of *Sordariomycetidae*	Iwarancusa grass (*Cymbopogon jwarancusa*)	Desert; between 27°42′0″ and 29°45′0″ N and 69°52′0″ and 75°24′0″ E—the southern part of Bahawalpur, Punjab, Pakistan	[32]
*Trichoderma harzianum*	Orchard grass (*Dactylis glomerata*)	Rongchang forage germplasm fields, Southwest University; 105°17′0″–105°44′0″ E, 29°15′0″–29°41′0″ N—Chongqing, China	[33]
*Trichoderma koningii*	Ginger (*Zingiber officinale*)	16°27′31″ N, 102°49′11″ E—Khon Kaen, Thailand	[22]

**Table 2:** Productions of IAA by EFs. NR = not reported.

Species	IAA Concentration (μg/mL)		Used Measurement Technique	Reference
	Without Trp	Supplemented with Trp		
*A. alternata*	40.12 ± 8.59	19.24 ± 14.88	HPLC	[28]
*A. sorghi*	NR	235.33 ± 7.51	colorimetric assay	[25]
*A. niger*	NR	60.04 ± 2.22	colorimetric assay	[21]
*C. fructicola*	NR	1205.58 ± 151.89	HPLC	[26]
*Colletotrichum gloeosporioides*	NR	243.56	colorimetric assay	[36]
*C. geniculata*	25.96	232.80	HPLC	[27]
*Paecilomyces formosus*	10.2 ± 1.21	NR	HPLC	[37]
*P. commune*	81.30 ± 0.30	192.10 ± 4.04	colorimetric assay	[31]
*P. commune*	NR	183.33 ± 7.53	colorimetric assay	[25]
*P. crustosum*	NR	63.5	colorimetric assay	[30]
*Sordariomycetidae* sp.	NR	1638	colorimetric assay	[32]
*T. harzianum*	5.44 ± 1.20	NR	colorimetric assay	[33]
*T. harzianum*	10.46 ± 2.40	NR	colorimetric assay	[33]
*T. harzianum*	4.87 ± 0.67	NR	colorimetric assay	[33]
*Tulasnella* sp.	NR	155.63	colorimetric assay	[36]

**Table 3:** IAA biosynthesis pathways of endophytic fungi.

Species	Pathway	References
*A. alternata*	IPA, IAM	[49]
*Alternaria* sp.	IPA, TSO	[49]
*Alternaria tenuissima*	IAM, TSO, IPA	[49]
*Camarosporidella moricola*	IPA	[49]
*C. gloeosporioides*	IAM	[3,50]
*Colletotrichum fruticola*	IAM	[51]
*Colletotrichum acutatum*	IAM	[51,52]
*Didymella glomerata*	IPA, TAM, IAM	[50]
*Didymella* sp.	IPA, TAM	[49]
*Erwinia herbicola*	IAM, IPA	[51]
*Fusarium sporotrichioides*	IAM, IPA	[49]
*Fusarium redolens*	IPA, IAM	[49]
*Fusarium armeniacum*	IAM	[49]
*Fusarium oxysporum*	TAM, IPA, IAM	[49]
*Fusarium verticillioides*	TSO, IAM, TAM, IPA	[49]
*Fusarium chlamclosporum*	TSO, IPA	[49]
*Fusarium tricinctum*	IAM	[49]
*Fusarium incarnatum*	IPA, IAM, TAM	[49]
*Fusarium proliferatum*	IPA, IAM	[49]
*Fusarium* sp.	IPA, TAM, IAM	[49,53]
*Fusarium graminearum*	IAN	[54,55]
*C. gloeosporioides f.* sp. *aeschynomene*	IAM	[51]
*Gibberella fujikuroi*	IPA	[41]
*Leptosphaeria maculans*	IPA	[56]
*Metarhizium robertsii*	TAM	[55]
*Paraphoma chrysanthemicola*	IPA	[49]
*Piriformospora indica*	IPA	[57]
*Sclerotinia sclerotiorum*	IAN	[54,55]
*Rhizobium* sp.	IAM	[58]
*Rhizoctonia* *solani*	IPA	[41]
*Ustilago maydis*	IPA	[59,60]
*Venturia nashicola*	IPA	[41]

## Data Availability

No new data were created or analyzed in this study. Data sharing is not applicable to this article.

## Abbreviations

ACC, 1-aminocyclopropane-1-carboxylic acid; IAM, indole-3-acetamide; IAA, indole-3-acetic acid; IAAld, indole-3-acetaldehyde; IAN, indole-3-acetonitrile; IAOx, indole-3-acetaldoxime; ILA, indole-3-lactic acid; IPA (IPyA), indole-3-pyruvic acid; NR, not reported; PDB, potato dextrose broth; Trp, L-tryptophan; TAM, tryptamine; TOL/IOL, indole-3-ethanol; TSO, tryptophan side-chain oxidase.
